# Cholestane-3β,5α,6β-triol Induces Multiple Cell Death in A549 Cells via ER Stress and Autophagy Activation

**DOI:** 10.3390/md22040174

**Published:** 2024-04-13

**Authors:** Jiaxi Chen, Jieping Zhang, Lijuan Cai, Li Guo, Zhenyu Cai, Hua Han, Wen Zhang

**Affiliations:** 1 School of Medicine, Tongji University, 1239 Si-Ping Road, Shanghai 200092, China; 2 Ningbo Institute of Marine Medicine, Peking University, 56 Kang-Da Road, Ningbo 315832, China

**Keywords:** Cholestane-3β,5α,6β-triol, cell death, ER stress, autophagy, ROS, oxidative stress

## Abstract

Cholestane-3β,5α,6β-triol (CT) and its analogues are abundant in natural sources and are reported to demonstrate cytotoxicity toward different kinds of tumor cells without a deep probe into their mechanism of action. CT is also one of the major metabolic oxysterols of cholesterol in mammals and is found to accumulate in various diseases. An extensive exploration of the biological roles of CT over the past few decades has established its identity as an apoptosis inducer. In this study, the effects of CT on A549 cell death were investigated through cell viability assays. RNA-sequencing analysis and western blot of CT-treated A549 cells revealed the role of CT in inducing endoplasmic reticulum (ER) stress response and enhancing autophagy flux, suggesting a putative mechanism of CT-induced cell-death activation involving reactive oxygen species (ROS)-mediated ER stress and autophagy. It is reported for the first time that the upregulation of autophagy induced by CT can serve as a cellular cytotoxicity response in accelerating CT-induced cell death in A549 cells. This research provides evidence for the effect of CT as an oxysterol in cell response to oxidative damage and allows for a deep understanding of cholesterol in its response in an oxidative stress environment that commonly occurs in the progression of various diseases.

## 1. Introduction

Cholestane-3β,5α,6β-triol (CT) and its analogues are highly oxygenated steroids that abundantly present in natural sources, particularly in marine invertebrates like sponges and soft corals [1,2]. We have previously obtained a variety of the polyhydroxysterol analogues from soft corals collected from the South China Sea [3,4,5,6]. These metabolites are widely reported to demonstrate cytotoxicity toward different kinds of tumor cells without a deep probe into their mechanism of action. What is more, CT is found as oxysterol in humans and mammals. The oxygenation of cholesterol in cytochrome P450 regulates cholesterol balance [7]. The 5,6-double bond in ring B of cholesterol is susceptible to non-enzymatic oxidation via free radical and non-free radical reactions, leading to the formation of 7-ketocholesterol (7-KC), 7β-hydroxycholesterol (7β-OHC), and 5α,6α/5β,6β-cholesterol epoxides [8]. The 5,6-cholesterol epoxides are catalyzed by cholesterol-5,6-epoxide hydrolase (ChEH), a microsomal epoxide hydrolase, to form CT [9].

Oxysterols are known to associate with numerous major pathologies by modulating many key cellular processes [10,11,12]. Both 7-KC and 7β-OHC cause a specific form of cytotoxic activity defined as oxiapoptophagy, including oxidative stress and the induction of death by apoptosis associated with autophagy [13]. 7-KC activates the NADPH oxidase (Nox-4) via endoplasmic reticulum (ER) stress involving the inositol-requiring enzyme type 1 (IRE1)/Jun-NH_2_-terminal kinase (JNK)/activator protein 1 (AP-1) signaling pathway, which contributes to the overproduction of reactive oxygen species (ROS) [14]. These oxysterols also induce an increase in cytosolic Ca^2+^ concentration associated with mitochondrial depolarization, leading to the Bid cleavage and poly (ADP-ribose) polymerase (PARP) degradation, and the activation of caspase-3, -7, -8, and/or -9 [13,15,16]. During the oxysterol-induced smooth muscle cell apoptosis, the reversibility of mitochondrial conformational change rather than the swelling and rupture of the outer membrane suggests that the apoptotic cascade can be arrested before a point of no return [17].

CT is one of the most abundant and active auto-oxidative forms of cholesterol in mammals [18]. However, comparatively less information is available on the CT than those of 7-KC and 7β-OHC [13]. CT was reported for the first time in 2005 to cause increased ROS in CHO cells and was then found to associate with nitric oxide synthase and modulated cyclooxygenase-2 expression via the PI3K-Akt-eNOS-dependent pathway [19,20,21]. CT was also a determinant in the impairment of mitochondrial function [22]. It was able to induce an increase in intracellular Ca^2+^ and Ca^2+^-dependent ROS generation in marrow stromal cells and increase the calcifying nodule formation, calcium deposition, alkaline phosphatase activity, and the apoptosis of nodular cells in calcifying vascular smooth muscle cells [23,24]. An in vitro study suggested that CT is more potent than other oxysterols as an inducer of apoptosis [25]. In the course of our ongoing search for bioactive molecules from marine sources, CT was repeatedly obtained from the South China Sea gorgonians, showing a significant tumor cell growth-inhibitory activity against A549 cells [4]. In the present study, the effects of CT on A549 cell death were investigated, showing that CT could induce ER stress and autophagy in A549 cells by increasing the levels of ROS.

## 2. Results

### 2.1. Effect of CT on A549 Cell Death

**Figure 1:**
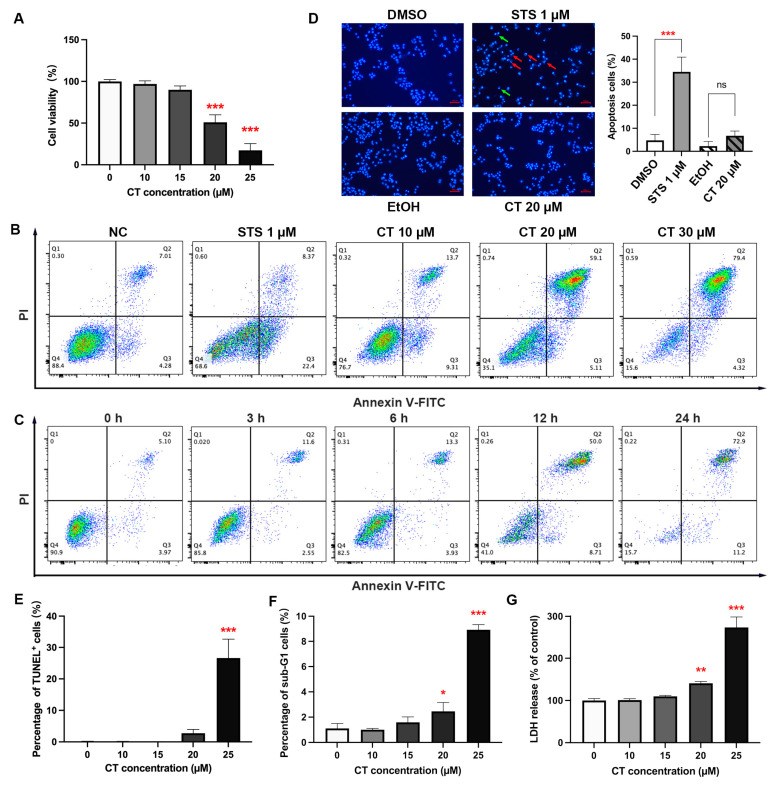
CT induced the cell death of human A549 cells. (**A**) CT inhibited the cell viability of human A549 cells. Cell viability was assessed using CCK-8 kit after the treatment of CT at different concentrations (10–25 µM) for 24 h. Statistical significance was determined using one-way ANOVA with Dunnett’s post-test, *n* = 3, *** *p* < 0.001 compared with the negative control (NC) group. (**B**) Typical diagram of annexin V/PI stain by flow cytometry. A549 cells were treated with different concentrations of CT for 24 h and subjected to annexin V and PI double staining. (**C**) Time-manner of typical diagram of annexin V/PI stain after treating with CT. A549 cells were treated with 20 µM CT for 3, 6, 12, 24 h and subjected to annexin V and PI double staining. (**D**) Detection of apoptosis using Hoechst 33342 staining in CT-treated cells. STS group showed many apoptotic cells with condensed (green arrows) and fragmented (red arrows) nuclei. The cells treated with 20 µM CT for 24 h showed a few apoptotic cells with condensed nuclei (Scale bar: 100 μm). Statistical significance was determined using one-way ANOVA with Tukey’s post-test in three different fields of view, *** *p* < 0.001 compared with the DMSO group, ns compared with ethanol (EtOH) group. (**E**) The TUNEL assay of A549 cells under CT treatment for 24 h. One-way ANOVA with Dunnett’s post-test was performed, *n* = 3, *** *p* < 0.001 compared with the control group. (**F**) The cell percentage of sub-G1 peak was determined following CT treatment for 24 h. One-way ANOVA with Dunnett’s post-test was performed, *n* = 3, * *p* < 0.05, *** *p* < 0.001 compared with the NC group. (**G**) CT increased LDH release in A549 cells. The assay was performed after the treatment of CT at different concentrations for 24 h. One-way ANOVA with Dunnett’s post-test was performed, *n* = 3, ** *p* < 0.005, *** *p* < 0.001 compared with the NC group.

CT is found to display a common cytotoxicity towards various cell lines, including A549, MG63 [4], HepG2, HEK293T, SH-SY5Y, N2a, and BV2 (Figure S1). The inhibition effect of CT on A549 cells was detected by Cell Counting Kit-8 (CCK-8) assay, as reported in our previous study [4]. CT inhibited the viability of A549 cells in a dose-dependent manner between 15 and 25 µM for 24 h, with the IC_50_ of 20.14 µM (Figure 1A and Figure S2). We used annexin V/propidium iodide (PI) analysis to identify the type of cell death induced by CT. According to flow cytometric results of CT-treated A549 cells, a significant increase in the proportion of annexin V^+^/PI^+^ cells could be observed under CT treatment with different concentrations or at different times (Figure 1B,C). Compared to the positive control (Figure S3), the staurosporine (STS) treatment group, the early stage of apoptotic cells (annexin V^+^/PI^−^ cells) under the CT treatment group did not increase significantly. It has been reported that CT could induce apoptosis in different types of cells [23,26,27], while our results demonstrated that CT mainly changed the permeability of the cell member. To verify the apoptosis-induced effect of CT, Hoechst 33342 staining, a terminal deoxynucleotidyl transferase-mediated dUTP nick end labeling (TUNEL) assay, and the detection of the sub-G1 peak in the DNA histogram were performed. Incubating A549 cells with STS, not the CT group, resulted in significant perinuclear chromatin condensation and nuclear fragmentation (Figure 1D). Similar results were observed in a TUNEL assay and sub-G1 peak proportion, where less than 30% of apoptotic cells were detected under a 25 µM CT treatment (Figure 1E,F). We also tried to detect the levels of cleaved-PARP and cleaved-caspase-3 by using a western blot (WB) analysis with STS as a positive control and cholesterol as an isotype control (Figure S4). The traces of WB bands for cleaved-PARP and cleaved-caspase-3 could be recognized only when taking long exposures on the films. This fact suggested that caspase-3 activation is not a dominant induction of cell death in response to CT. A lactate dehydrogenase (LDH) release assay was performed to examine the effect of CT on membrane integrity in A549 cells. The concentration-dependent cytotoxicity effect of CT was reproduced in the LDH assay (Figure 1G). These results demonstrated that CT inhibited A549 cell viability along with less than 30% of apoptotic cells occurring under CT exposure. This fact indicated that the cell death induced by CT was not limited to the way of apoptosis.

### 2.2. CT Activated the Response to ER Stress in A549 Cells

To gain a better understanding of the possible signaling processes involved in CT-induced cell death, we interrogated the transcriptome of A549 cells under CT treatment. We chose three different CT doses to represent different cellular stages: no cytotoxic concentration at 10 µM, sublethal concentration at 15 µM, and significant cytotoxic concentration at 20 µM. Firstly, RNA-sequencing (RNA-seq) was conducted to disclose the cell-signaling pathway that is involved in the CT-induced cell death in A549 cells.

**Figure 2:**
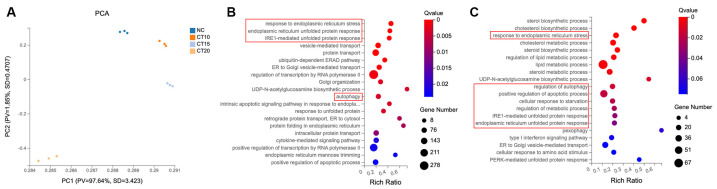
RNA-seq analysis of different concentrations of CT-treated A549 cells. (**A**) PCA analysis using most variable genes revealed the separation of four groups. PCA identifies the differences among each group, *n* = 3. (**B**) GO analysis of the upregulated gene clusters in CT 20 µM treatment groups. The function of Q value ≤ 0.05 was considered a significant enrichment. (**C**) GO analysis of the upregulated gene clusters in CT 15 µM treatment groups. ER stress and autophagy-related biological processes were highlighted in red boxes in B and C.

The principal component analysis (PCA) recapitulated the overall morphologic classification of the NC group and three CT-treatment groups (Figure 2A). The CT 20 µM treatment cohort was clearly separated from the other cohorts, indicating that there were many gene-expression differences between CT 20 µM and the other cohorts. The CT 10 µM treatment cohorts tended to be located closer to the NC cohort than CT 15 µM. This observation indicated a significant dynamic change in the gene transcription relevant to the CT-accumulation process.

The number of different expression genes (DEGs, fold change ± 1.5) in the CT 10, 15, and 20 µM treatment groups were 301 (224 upregulated DEGs, 77 downregulated DEGs), 1832 (1198 upregulated DEGs, 634 down-regulated DEGs), and 4679 (2405 upregulated DEGs, 2274 downregulated DEGs), respectively. To expand our understanding of CT’s functional role at the 20 µM concentration in A549 cells, we performed a Gene Ontology (GO) analysis to infer the biological pathways related to the 2405 upregulated genes (Figure 2B). The top three biological processes included the response to ER stress, ER unfolded-protein response, and IRE1-mediated unfolded-protein response. Response to ER stress also showed in the GO analysis results of CT 15 µM group (Figure 2C). As for the CT 10 µM treatment group, genes for processes of cholesterol biosynthetic and lipid metabolism were mainly affected, and no ER stress process occurred (Figure S5). These findings prompted the investigation of CT on the ER stress process.

**Figure 3:**
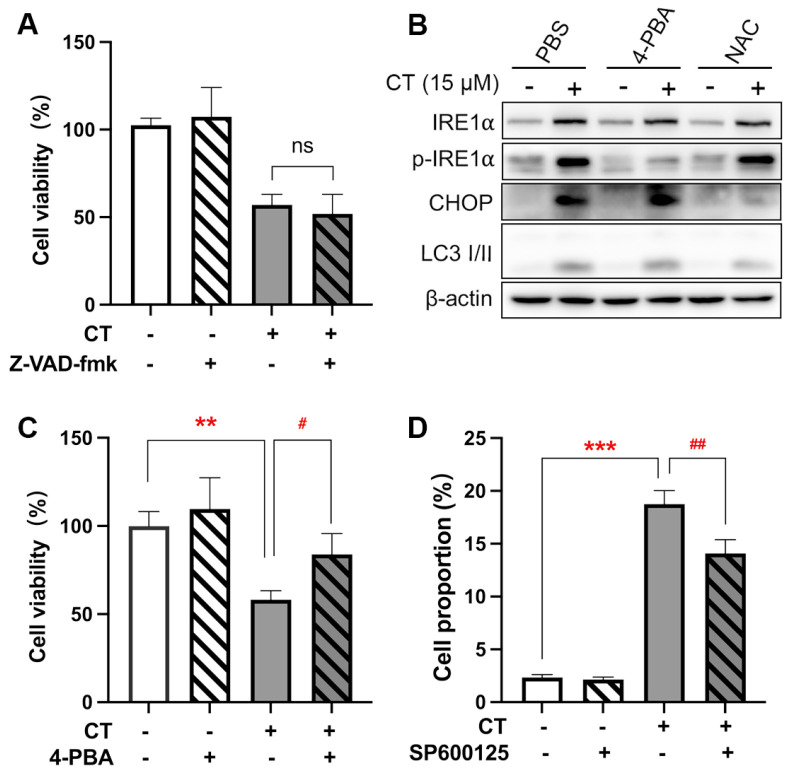
CT was associated with the ER stress process in A549 cells. (**A**) The effect of the pan-caspase inhibitor on CT-induced cell death. The cells were pre-treated with 20 µM Z-VAD-fmk for 1 h, then incubated with 20 µM CT for 24 h. Cell viability was assessed using a CCK-8 kit. One-way ANOVA with Tukey’s post-test was performed, *n* = 4, ns compared with CT group. (**B**) WB of ER stress-associated protein levels in A549 cell lines treated with CT. The cells were pre-treated with 1 mM 4-PBA or 1 mM NAC for 1 h, then incubated with CT at 15 µM for 24 h. (**C**) The effect of ER stress inhibitor on CT-induced cell death. The cells were pre-treated with 1 mM 4-PBA for 1 h, then incubated with 20 µM CT for 24 h. Cell viability was assessed using a CCK-8 kit. Statistical significance was determined using an unpaired *t*-test, *n* = 3. # *p* < 0.05 compared with the CT 20 µM group. ** *p* < 0.005 compared with the NC group. (**D**) Percentage of annexin V^+^/PI^+^ and annexin V^+^/PI^−^ cells after being treated with CT. A549 cells were pre-treated with 10 µM SP600125 for 1 h, then incubated with 20 µM CT for a further 24 h and subjected to annexin V and PI double staining. Statistical significance was determined using one-way ANOVA with Tukey’s post-test, *n* = 3. ## *p* < 0.005 compared with the CT group, *** *p* < 0.001 compared with the NC group.

ER stress response-associated gene expressions were the most enriched. ER-induced apoptosis occurs via three primary pathways, including the IRE1/apoptosis signal–regulating kinase 1 (ASK1)/JNK pathway, the caspase-12 kinase pathway, and the C/EBP homologous protein (CHOP)/DNA damage-inducible gene 153 (GADD153) pathway [28]. Firstly, we observed that the pan-caspase inhibitor, Z-VAD-fmk, did not affect CT-induced cytotoxicity in A549 cells (Figure 3A). The expression of caspase-12 was therefore not further evaluated. We then measured the additional two biomarkers of the ER stress-mediated apoptosis pathway, p-IRE1α and CHOP [29], to test the activation of ER stress response in A549 cells after the treatment of CT by WB. The increased expression of p-IRE1α and CHOP indicated that CT activated a response to ER stress (Figure 3B and Figure S6). The IRE1α phosphorylation induced by CT was found to be attenuated by the ER stress inhibitor, sodium 4-phenylbutyrate (4-PBA) [30]. Similar results were observed in the cell viability assay (Figure 3C). There was a significant difference between the 4-PBA-pre-treatment group and the CT-treatment group, whereas no significant statistical difference was found between the 4-PBA pre-treatment and NC groups. Interestingly, the 4-PBA could not reverse the upregulated CHOP levels (Figure 3B), suggesting that CT did not increase the CHOP expression through the ER stress response. Since IRE1α is upstream from the JNK that mediated ER stress-induced cell death [31], we evaluated the effect of SP600125 as a JNK inhibitor on CT-induced cell death through an annexin V/PI staining experiment. As shown in Figure 3D, the proportion of CT-induced dead cells (annexin V^+^/PI^−^ and annexin V^+^/PI^+^ cells) was decreased when treated with SP600125. The facts revealed that CT-induced cell death was associated with IRE1α/JNK-mediated ER stress response.

### 2.3. CT Enhanced Autophagy Flux in A549 Cells

**Figure 4:**
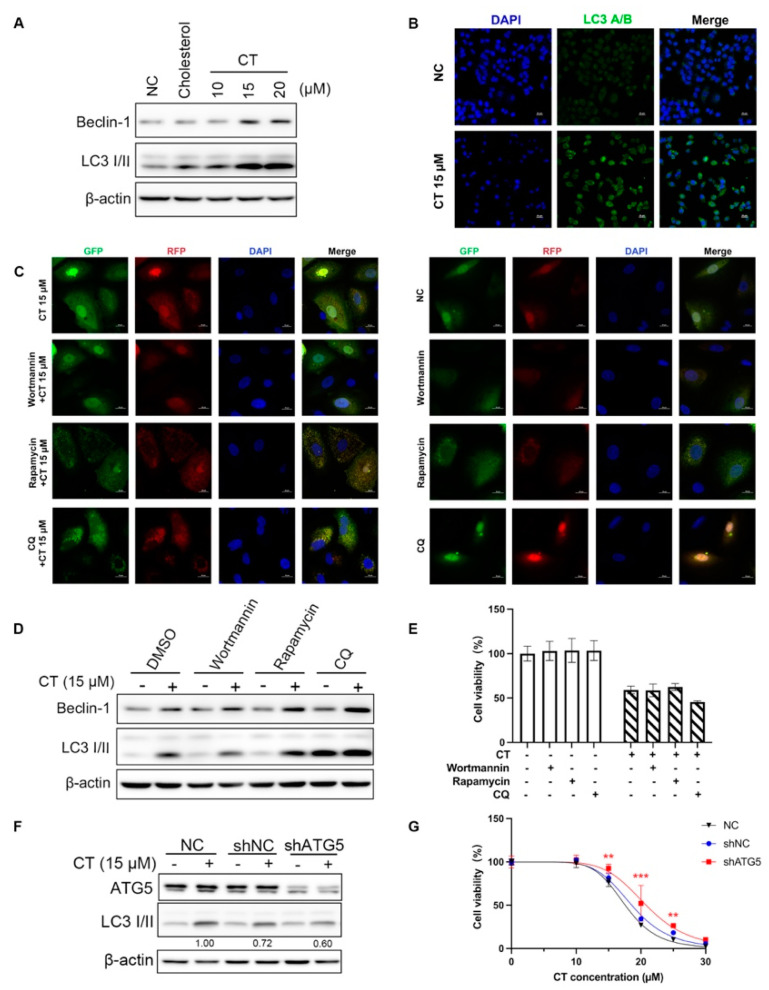
Autophagy occurred in A549 cells in the presence of CT. (**A**) WB of autophagy markers levels in A549 cell lines treated with a series dose of CT. The cells were treated with 10, 15, and 20 µM CT for 24 h, regarding cholesterol (20 µM) as an isotype control. β-actin was used as a loading control. (**B**) Immunofluorescence detection of cytoplasmatic LC3 levels in A549 cells exposed to 15 µM CT for 24 h. Upper row: negative control cells. Lower row: CT 15 µM-treated cells. Left column: DAPI-labeled nuclei. Middle column: anti-LC3 antibody conjugated FITC visualized LC3 puncta. Right column: two-color overlay showed diffuse LC3 cytoplasmic puncta (Scale bar: 20 µm). (**C**) Presence of autophagosomes (yellow) in mRFP-GFP-LC3 adenovirus-transfected cells after 15 µM CT treatment with or without autophagy-interfering reagents (Scale bar: 20 μm). (**D**) WB of LC3 levels under the presence of three types of autophagy-interfering reagents and 15 µM CT. The cells were pre-treated with wortmannin (50 nM), rapamycin (2 µM), or CQ (10 µM) for 1 h, then incubated with 15 µM CT for 24 h. (**E**) The effect of autophagy-interfering reagents on CT-induced cell death. The cells were pre-treated with 50 nM wortmannin, 2 µM rapamycin, or 10 µM CQ for 1 h, then incubated with 20 µM CT for 24 h. Cell viability was assessed using a CCK-8 kit. Statistical significance was determined using two-way ANOVA with Dunnett’s post-test, *n* = 3. (**F**) WB of LC3 levels in *Atg5*-silenced A549 cells. The cells were incubated with 15 µM CT for 24 h. (**G**) The cytotoxicity of CT in *Atg5*-silenced A549 cells. The shAGT5-transfected A549 cells were incubated with a series dose of CT for 24 h. Cell viability was assessed using a CCK-8 kit. Statistical significance was determined using two-way ANOVA with Dunnett’s post-test, *n* = 3. ** *p* < 0.005, *** *p* < 0.001, shATG5 cells compared with the NC group.

The RNA-seq analysis revealed that autophagy may occur in CT-treated A549 cells. Autophagy-related genes were enriched in the upregulated DEG clusters of both CT 15 and 20 µM treatment groups (Figure 2B,C). However, it has not been reported that CT can induce autophagy. To determine whether CT induces cell death through autophagy in A549 cells, we analyzed the corresponding changes in autophagy-related proteins in A549 cells after being treated with CT at 15 µM. Beclin-1 displays an essential role in autophagy nucleation and regulates autophagy positively [32]. The conversion of microtubule-associated protein 1 light chain 3 (LC3) I into II is widely used as a marker for autophagosome formation [33]. As evidenced by WB, CT significantly increased the expression of the crucial autophagy biomarkers at a dose-dependent manner in A549 cells, including Beclin-1 and LC3 II (Figure 4A). The clustering of LC3 was also detected by immunofluorescence in A549 cells with sub-lethal CT concentrations (Figure 4B).

To confirm the underlying mechanism, we compared the changes in Beclin-1 and LC3 with the pre-treatments of hydrochloroquine (CQ), wortmannin, and rapamycin. The LC3 II levels were decreased by wortmannin, an autophagy inhibitor, and increased by rapamycin, which controls the autophagy-negative regulators of mTOR localization to attenuate autophagy flux (Figure 4C,D). CQ is a lysosomal protease inhibitor, resulting in LC3 II accumulation within lysosomes, leading to lysosome neutralization [34]. As shown in Figure 4C, the number of total puncta was elevated after CT treatment, while CQ treatment further enhanced autophagosome accumulation (indicated by yellow puncta) in A549 cells treated with CT. CQ treatment elicited a further accumulation of LC3 II in WB (Figure 4D). The accumulation of autophagosomes indicated either the upregulation of autophagic flux (i.e., autophagosome formation) or the blockade of autophagic degradation [35]. These observations suggest that CT promotes the accumulation of both autophagosomes and autolysosomes in A549 cells, inferring propelled autophagic flux. To investigate the role of autophagy in CT-induced cell death, we measured the cell viability in A549 cells treated with these autophagy agonists or inhibitors before CT exposure. However, we did not observe any alleviation of cell viability in the autophagy-interfering reagent pre-treatment group (Figure 4E). Then, we silenced endogenous *Atg5* with shRNA in A549 cells to evaluate whether autophagy mediates CT-induced cell death. *Atg5* is one of the most targeted genes in autophagy gene-editing assays. Knocking down or knocking out *Atg5* can result in the downregulation or total inhibition of autophagy [36]. The LC3 II levels were decreased in *Atg5* knockdown A549 cells (Figure 4F). The decreased cell viability induced by CT was modestly reversed by silencing *Atg5* (Figure 4G). These results indicate that CT-induced autophagy can promote cell death in A549 cells.

### 2.4. CT-Induced ROS Generation Triggered ER Stress and Autophagy

**Figure 5:**
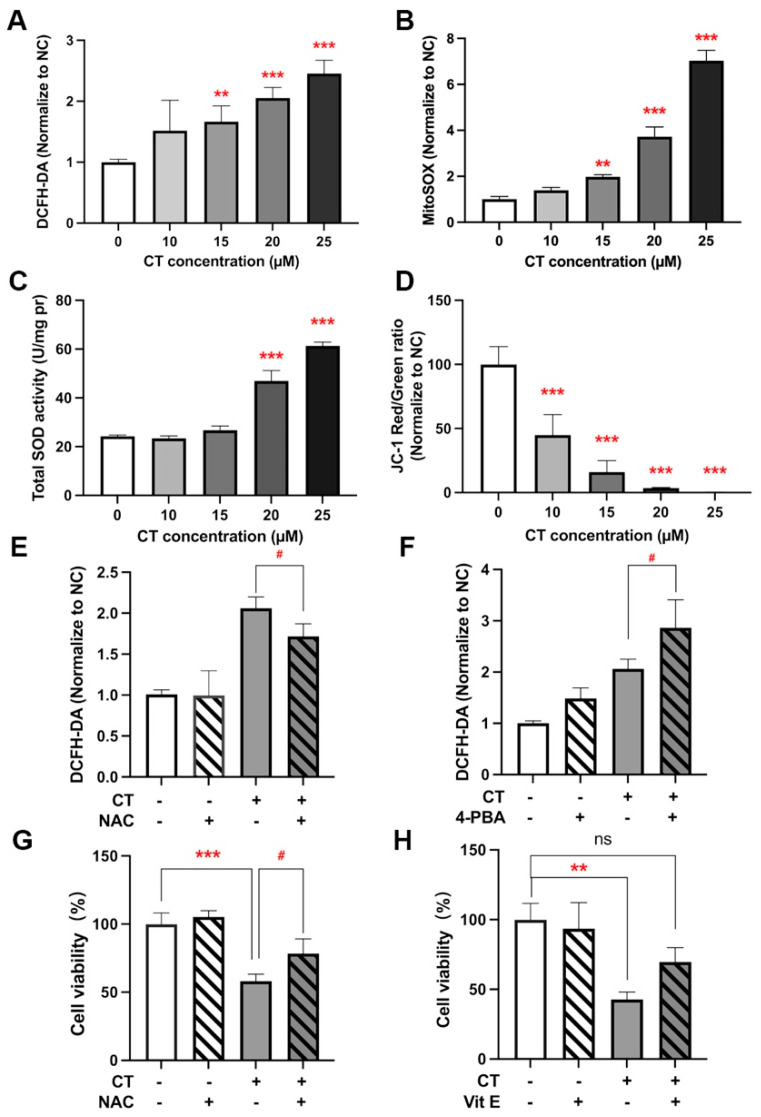
ROS generation activated ER stress and reduced cell viability. (**A**) Analysis of ROS levels in A549 cells under CT exposure. A549 cells were treated with a series of doses of CT for 24 h. Then, cells were harvested and stained with DCFH-DA. Statistical significance was determined using one-way ANOVA with Dunnett’s post-test, ** *p* < 0.005, *** *p* < 0.001 compared with the NC group. (**B**) Analysis of MitoSOX levels in A549 cells under CT exposure. Cells were harvested and stained with MitoSOX after being treated with a series of doses of CT for 24 h. Statistical significance was determined using one-way ANOVA with Dunnett’s post-test, ** *p* < 0.005, *** *p* < 0.001 compared with the NC group. (**C**) Total SOD activity of A549 cells under CT exposure. Statistical significance was determined using one-way ANOVA with Dunnett’s post-test, *** *p* < 0.001 compared with the NC group. (**D**) The mitochondrial membrane potential of A549 cells was evaluated by using JC-1 staining. Statistical significance was determined using one-way ANOVA with Dunnett’s post-test, *** *p* < 0.001 compared with the NC group. (**E**) The effect of NAC on CT-induced ROS upregulation by flow cytometer. A549 cells were incubated with 1 mM NAC for 1 h before incubating with 20 µM CT for a further 24 h. Statistical significance was determined using two-way ANOVA with Šídák’s post-test, # *p* < 0.05 compared with the CT group. (**F**) The effect of 4-PBA on CT-induced ROS upregulation by flow cytometer. A549 cells were incubated with 1 mM 4-PBA for 1 h before incubating with 20 µM CT for a further 24 h. Statistical significance was determined using two-way ANOVA with Šídák’s post-test, # *p* < 0.05 compared with the CT group. (**G**,**H**) The effect of antioxidants on CT-induced cell death. The cells were pre-treated with 1 mM NAC or 10 µM Vit E for 1 h, then incubated with 20 µM CT for a further 24 h. Cell viability was assessed using a CCK-8 kit. Statistical significance was determined using one-way ANOVA with Tukey’s post-test, # *p* < 0.05 compared with the CT 20 µM group. ** *p* < 0.005, *** *p* < 0.001, and ns compared with the NC group.

In 2005, it was reported for the first time that CT could induce genotoxicity in a ROS-dependent manner [19]. CT was then reported to induce mitochondrial ROS production and subsequently oxidative damage in vascular cells, leading to mitochondrial dysfunction, which suggests a putative mechanism of apoptosis activation by oxysterols [22]. According to our results, CT-induced cell death is associated with ER stress and autophagy, as described above. To further investigate the role of CT-induced ROS generation in ER stress response, autophagy, and cell death, we evaluated ROS levels with DCFH-DA and MitoSOX in CT-treated A549 cells. DCFH-DA is a cytosolic ROS sensor and MitoSOX is a mitochondrial ROS molecular probe. These flow cytometric assays showed that CT remarkably raised the level of ROS in intracellular and mitochondria concentration dependently (Figure 5A,B). A concentration-dependent increase in total superoxide dismutase (SOD) activity was also detected in A549 cells after CT treatment (Figure 5C). We evaluated mitochondrial function by detecting mitochondrial membrane potential (△Ψm), which could be decreased via CT (Figure 5D).

Next, we explored the possible relationship between ROS and ER stress by using antioxidant N-acetyl-L-cysteine (NAC). The increased ROS levels were modestly alleviated by NAC (Figure 5E). Meanwhile, NAC could also modestly attenuate the upregulation of CHOP and p-IRE1α expression based on the WB analysis (Figure 3B and Figure S6), suggesting that CT activated ER stress through ROS-related pathways. The intracellular ROS levels could not be reduced via 4-PBA even though p-IRE1α could be attenuated in the cotreatment (4-PBA + CT) group (Figure 3B and Figure 5F). Taken together, the above data indicated that CT-induced ROS generation contributed to the IRE1α-associated ER stress response in A549 cells.

Additionally, we further verified the role of ROS in autophagy regulation by WB. NAC could effectively reduce the LC3 II expression, whereas 4-PBA could not significantly change the levels of LC3 II, suggesting that the increase in autophagy flux mainly results from the increased ROS (Figure 3B and Figure S6).

To determine whether the CT-induced cytotoxicity was related to the generation of ROS, the effect of NAC and vitamin E (Vit E) on cell viability was investigated by CCK-8. NAC or Vit E treatment could improve cell viability. However, no significant statistical difference was observed between the cotreatment (CT + NAC and CT + Vit E) and NC groups (Figure 5G,H). These findings indicate that CT can induce ROS generation both intracellularly and in mitochondria, and the increased ROS levels then activate the ER stress response and autophagy flux, leading to the cell death of A549.

## 3. Discussion

Polyhydroxylated steroids are exist ubiquitously in natural sources. They are also widespread in mammals which can participate in the development of various diseases, such as neurodegenerative diseases [37,38], atherosclerosis [39], and nonalcoholic steatohepatitis [40]. CT was also reported to accumulate in cancers, e.g., lung cancer, breast cancer, and colon cancer [41,42,43]. CT and the analogues that we obtained from the South China Sea gorgonians were identified as apoptosis inducers in A549 and MG63 cells [4]. However, some research on CT-induced apoptosis lacked direct evidence. The report of CT inducing apoptosis in prostate cancer cells was only detected by a TUNEL assay [44]. Although oligonucleosomal cleavage, and the changed expression of apoptosis-related proteins in MMNK-1 cells suggested that CT-induced apoptosis was associated with mitochondrial-dependent mechanisms, there was no observation of any increased number of early apoptotic cells [45]. It was reported that CT could induce a significant LDH leakage in ECV-304 cells, but apoptosis induced by CT was not detected by flow cytometry, fluorescence microscope, and agarose gel electrophoresis [46]. According to flow cytometric results of CT-treated A549 cells in our research, a significant increase in the proportion of annexin V^+^/PI^+^ cells could be observed under CT treatment with different concentrations or at different times. This led to several classic experiments to verify the apoptosis-induced effect of CT, including Hoechst 33342 staining, the detection of a sub-G1 peak, and a TUNEL assay. The experiments showed that not more than 30% of cells underwent apoptosis under CT treatment. Additional modes of CT-induced cell death must exist for further investigation.

To gain a more comprehensive understanding of the processes, RNA-seq analysis was performed to identify potential regulatory factors. According to the GO analysis, ER stress response-associated gene expressions were the most enriched. ER stress is known to mediate different modes of cell death, including autophagy, apoptosis, ferroptosis, and pyroptosis [47,48]. We detected the expression of p-IRE1α and CHOP increased in CT-treated A549 cells. Cotreatment with 4-PBA completely reversed the expression of p-IRE1α but not the CHOP levels. CHOP was not only regulated by ER stress [28], but was also proposed to be the main transcription factor that conveys the specificity of the mitochondrial-stress response in the context of mitochondrial dysfunction [49]. Taken together with the characterization of mitochondrial dysfunction by measuring △Ψm and mitochondrial ROS levels, we presume that CT upregulated CHOP expression through ROS-related pathways, but not the ER stress pathway. The certain reversal effect of 4-PBA and SP600125 on cell death indicated that the CT-activated ER stress response mediated the cell death in A549 cells via the IRE1α/JNK pathway.

The RNA-seq analysis also revealed that autophagy may occur in CT-treated A549 cells. Numerous studies were reported for 7-KC and 7β-OHC regarding their role in inducing autophagy [13], but it has never been reported that CT can induce autophagy so far. Autophagy often accompanies cell death, yet autophagy-dependent cell death is highly contextual [50,51]. CT contributed to autophagy flux in a concentration-dependent manner. Wortmannin, rapamycin, and CQ changed LC3 II expression levels, but no significant cell viability change was observed. To further confirm the effect of autophagy in CT-induced cell death, we silenced *Atg5* expression; the activation of autophagy was correspondingly downregulated and the cell viability of A549 cells under CT treatment was reversed. These results demonstrated that autophagy was a promotor of CT-induced cell death, which is different from the protective role of autophagy induced by 7-KC and 7β-OHC [52].

**Figure 6:**
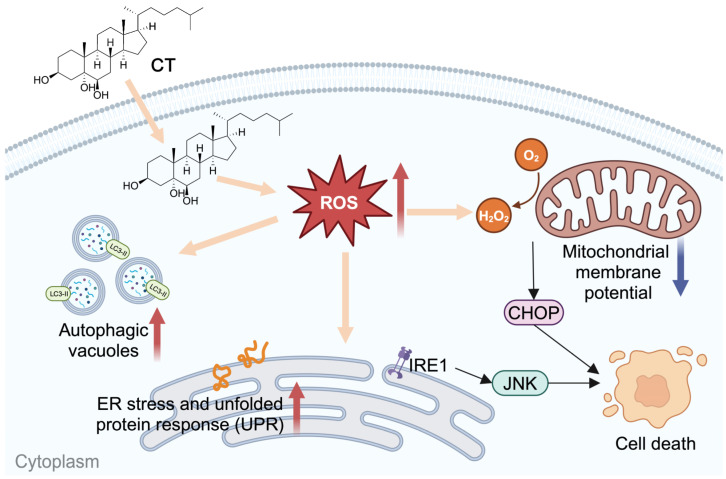
Scheme of CT effects on A549 cell lines.

CT was reported to induce the generation of ROS [22], which is an important factor in inducing ER stress and autophagy. Thus, we evaluated mitochondrial function by detecting △Ψm. The ROS levels of intracellular and mitochondrial, and total SOD activity were found to increase dose-dependently under CT treatment in A549 cells. Intracellular ROS induced by CT were slightly decreased by NAC treatment. At the same time, we observed that the p-IRE1α, CHOP, and LC3 II expression, as well as cell viability, had been improved under antioxidant protection. Collectively, the present results suggest that CT-stimulated ROS generation in both intracellular and mitochondria activates ER stress and autophagy, which subsequently leads to the cell death of lung-cancer cells (Figure 6).

In conclusion, our study provided a systems-level insight into the potential signaling pathways being affected by CT in A549 cells. This is the first report of CT inducing death-promoting autophagy. This research proposes a hypothesis that the autoxidized cholesterol in high-cholesterol microenvironments plays a key role in accelerating the pathogenesis of diseases. Excess cholesterol can be autoxidized to oxysterols during oxidative stress conditions. Subsequently, the accumulation of non-enzymatic oxysterols facilitates the development and progression of diseases by impairing mitochondrial function and redox balance [53]. The hypothesis is in agreement with the report that cholesterol enrichment accelerates and worsens the pathogenesis of neurodegenerative diseases by enhancing the mitochondrial oxidative stress elicited by Aβ [54]. Further investigation is needed for a better understanding of the mechanism. Meanwhile, the signaling pathways activated by 7-KC and 7β-OHC are highly conserved from different cell types and seem to be independent of the species considered [52], so it is worthy of more in-depth research on the cellular mechanism modulated by oxysterols, including 7-KC, 7β-OHC, 5α,6α/5β,6β-cholesterol epoxides, and CT as well.

## 4. Materials and Methods

Materials. CT (Avanti Polar Lipids, Inc., Birmingham, AL, USA) was dissolved in ethanol and stored at −20 °C. STS and cholesterol were acquired from Selleck (Houston, TX, USA). 4-PBA, NAC, Vit E, SP600125, wortmannin, rapamycin, and CQ were purchased from MedChemExpress (Monmouth Junction, NJ, USA). Z-VAD-fmk, and puromycin dihydrochloride were purchased from Beyotime (Shanghai, China). CHOP (L63F7) mouse mAb (CAT#2895), Beclin-1 (D40C5) rabbit mAb (CAT#3495), LC3A/B (D3U4C) XP^®^ rabbit mAb (CAT#12741), and β-actin rabbit mAb (CAT#4970) were purchased from Cell Signaling Technology (Beverly, MA, USA). Recombinant anti-IRE1 (phospho S724) antibody (CAT#R26310) and APG5L rabbit pAb (CAT#381320) were purchased from Zen-bioscience (Chengdu, China). Goat anti-rabbit IgG, HRP-linked antibody (CAT#31460) was purchased from Invitrogen (Carlsbad, CA, USA). Goat anti-rabbit IgG-FITC (CAT# abs20023) was purchased from Absin Bioscience (Shanghai, China). Goat anti-mouse IgG, HRP-linked antibody (CAT#A0216) was purchased from Beyotime (Shanghai, China).

Cell Culture. A549, HepG2, HEK293T, SH-SY5Y, N2a, and BV2 were cultured in Dulbecco’s Modified Eagle Medium (DMEM, Procell, Wuhan, China), supplemented with 10% FBS (Viva Cell Biotechnology, Denzlingen, Germany) and 1% penicillin-streptomycin (Gibco, Carlsbad, CA, USA). The cultures were incubated at 37 °C with 5% CO_2_.

Cytotoxicity Assay. Cells were seeded on a 96-well plate at a density of 8000 cells per well and incubated overnight. Inhibitors were pre-treated for 1 h before incubating cells with CT for 24 h. Cell viability was assessed by using CCK-8 reagent (DojinDo, Nanjing, China). The absorbance was measured at 450 nm with a microplate absorbance reader (Infinite F50, TECAN, Männedorf, Switzerland) after incubation at 37 °C for 1 h.

Total LDH Release Assay. Cell integrity was assessed by quantifying LDH released into the culture media upon plasma-membrane disruption. The assay was performed using the LDH Cytotoxicity Assay Kit (Beyotime, Shanghai, China) following the manufacturer’s instructions. The absorbance was measured at 492 nm with 620 nm reference absorbance. LDH Release (% of control) = Sample Absorbance/Control Absorbance × 100%.

Apoptosis Assay by Flow Cytometry. Flow cytometry was performed with annexin V-FITC/PI Cell Apoptosis Detection Kit (Beyotime, Shanghai, China) to detect apoptosis. Cells were seeded on a 24-well plate at a density of 70,000 cells per well. After the treatments, cells were collected with trypsin (Gibco, Carlsbad, CA, USA), centrifuged at 300× *g* at 4 °C for 5 min, and washed twice with cold PBS. Cells were resuspended in 195 µL cold binding buffer. Then, 5 µL annexin V-FITC and 10 µL PI were added to cell suspensions and incubated in the dark at room temperature for 10 min. The cells were analyzed by flow cytometry (Becton Dickinson, Franklin Lakes, NJ, USA).

Hoechst 33342 Staining. For the detection of apoptosis, Hoechst 33342 (Beyotime, Shanghai, China) staining was used to visualize the apoptotic nucleus. After being washed with PBS, cells cultured in a 24-well plate were stained with 5 µg/mL Hoechst 33342 in PBS at 37 °C for 30 min. Apoptotic cells were characterized by the condensed or fragmented nuclei, as visualized using a fluorescence microscope (CKX53SF; Olympus, Tokyo, Japan).

TUNEL Assay. We used TUNEL assay (Beyotime, Shanghai, China) to detect DNA fragmentation in cells. Briefly, cells were fixed with 4% formaldehyde in PBS for 30 min at room temperature (RT), then permeabilized in 0.3% Triton X-100 solution in PBS for 5 min at RT after washing twice in PBS. TUNEL assay was then performed according to the manufacturer’s instructions. The cells were analyzed by flow cytometry.

Analysis of Double-stranded Breaks of DNA. DNA damage was determined by flow cytometry, based on the formation of sub-G1 peaks of DNA as follows. Cells were harvested and washed with ice-cold PBS and fixed with precooled 70% ethanol in PBS at 4 °C overnight. Cells were washed with PBS, then incubated with PI/RNase A staining reagent (Beyotime, Shanghai, China) at 37 °C for 30 min. Cells should be analyzed using a FACS-Calibur cytometer.

△Ψm Assay. We used JC-1 stain to detect △Ψm. Cells were harvested and washed twice with PBS and suspended in 500 µL PBS with JC-1 (MedChemExpress, Monmouth Junction, NJ, USA) at 2 μM. Keep the samples at 37 °C, 5% CO_2_ for 30 min. Cells were then analyzed immediately with the flow cytometer, typically equipped with a 488 nm and 561 nm argon laser. The results were calculated as MFI_Red_/MFI_Green_ × 100%.

Detection of Intracellular ROS. The production of intracellular ROS in cells after being treated with CT was measured by using DCFH-DA (Beyotime, Shanghai, China). Cells were harvested and washed twice with PBS and incubated with DCFH-DA at a final concentration of 10 μM to suspended cells. After being incubated at 37 °C for 30 min, cells were measured immediately with the flow cytometer. The results were calculated as MFI_exp_/MFI_NC_ × 100%.

Mitochondrial Superoxide Assays. Cells were harvested and washed twice with PBS. To quantify the average cellular integrated density of MitoSOX fluorescence, we incubated 500 nM MitoSOX (Thermo Fisher Scientific, Waltham, MA, USA) at 37 °C for 30 min. After being washed with PBS twice, cells were measured immediately with the flow cytometer. The results were calculated as MFI_exp_/MFI_NC_ × 100%.

RNA-seq. For RNA-seq analysis, A549 cells used TRIzol reagent (Invitrogen, Carlsbad, USA) to collect cells after treating cells with different concentrations of CT for 24 h. Later, all the samples were sent to BGI Corporation (Shenzhen, China) for further RNA-seq detection and analysis via BGISEQ-500 sequencer. Bioinformatics Workflow including data filtering, mapping transcript prediction, differential gene expression analysis, and GO and Pathway analysis were performed on the Dr. Tom network platform of BGI (http://report.bgi.com, accessed on 14 March 2023). Details can be provided upon request. Overall, we obtained an average of 1.18 G clean reads, with an average genome mapping rate of 93.27%. The total gene number identified was 17,814 genes.

Western Blot. Cell pellets were lysed in RIPA buffer (Beyotime, Shanghai, China) containing 1× general protease and phosphatase inhibitor cocktail (Absin Bioscience, Shanghai, China). Total protein was measured by the BCA protein assay (Beyotime, Shanghai, China) as per the manufacturer’s instructions. An equal amount of protein was loaded on 8–12% SDS–PAGE gels and electro-transferred to PVDF membranes. Blots were blocked in 5% skim milk (Becton Dickinson, Franklin Lakes, NJ, USA) or 5% BSA (Sigma-Aldrich; Merck KGaA, Darmstadt, Germany) in 0.05% TBST at RT for 2 h and then incubated with primary antibodies (1:1000) diluted in Primary Antibody Dilution Buffer (Abclonal, Woburn, MA, USA) at 4 °C overnight. After being washed three times with 0.05% TBST, the membranes were incubated with Goat anti-rabbit IgG (1:20,000) at RT for 1 h. Band signals were visualized on an Amersham Imager 680 (GE HealthCare Life Sciences, Marlborough, MA, USA) by using BeyoECL Star Kit (Beyotime, Shanghai, China). Band intensities were quantified by ImageLab processing system. β-Actin was used as reference control to normalize the detected proteins.

Immunofluorescence. The amount of LC3, an indicator of autophagosomes, was detected following a standard procedure of immunofluorescence. Firstly, cells were fixed with a 4% (*w*/*v*) paraformaldehyde solution for 15 min. Subsequently, the cells were permeabilized with 0.5% Triton X-100 in PBS for 10 min, then blocked with 1% BSA-PBS at RT for 2 h. The cells were washed three times with PBS between each step. The glass coverslips with A549 cells were incubated with LC3A/B (D3U4C) XP^®^ rabbit monoclonal antibody (1:200) at 4 °C overnight and subsequently with the corresponding FITC-linked goat anti-rabbit secondary antibody (1:200) at RT for 1 h. Finally, the cells were counterstained with DAPI (Beyotime, Shanghai, China) and sealed with antifade mounting medium (Beyotime, Shanghai, China). Fluorescence images were visualized with a Nikon Eclipse microscope system (Tokyo, Japan).

Establishment of mRFP-GFP-LC3 A549 Cell Line. To visualize autolysosomes/autophagosomes under a laser confocal microscope, A549 cells were transfected with adenovirus mRFP-GFP-LC3 (HanBio Technology, Shanghai, China). Briefly, after the cells were seeded and reached 50% confluence, they were transfected with mRFP-GFP-LC3 adenovirus according to the manufacturer’s instructions. The medium was renewed 6–8 h after transfection to remove extra adenovirus. CT treatments were conducted at least 48 h after transfection. After CT treatment, the transfected cells were fixed with 4% paraformaldehyde and observed under a Nikon Eclipse microscope system.

RNA Interference by Lentivirus-Derived shRNA. pLKO.1-puro plasmid containing shRNA targeting ATG5 was purchased from Tsingke Biotechnology (Beijing, China). HEK293T cells were co-transfected with pLKO.1-puro plasmid and packaging plasmids by Lipofectamine 2000 transfection reagent (Invitrogen, Carlsbad, CA, USA). Viral particle-containing supernatants were harvested 24 and 48 h later. A549 cells were infected with the lentivirus vector with 8 μg/mL polybrene (Beyotime, Shanghai, China) for 15 h. After 24 h of culture, the medium was added to 1 µg/mL puromycin dihydrochloride for 3 days. After all the cells in the blank group died, the normal medium was replaced to terminate the screening and continue to expand the culture.

Detection of Total SOD Activity. Cells were lysed by 1% Triton X-100 in PBS. The supernatants were used to measure total SOD activity by a SOD Assay kit (Dojindo, Nanjing, China) according to the manufacturer’s recommendations.

Statistics Analysis. The differences between each group were analyzed using GraphPad Prism software (version 9.3.1, GraphPad Software, Boston, MA, USA). For statistical analyses, one-way ANOVA or two-way ANOVA were used for multiple comparisons. Data were presented as mean ± SD. Statistical significance was considered at *p* < 0.05.

## Data Availability

The original data presented in the study are included within the article or Supplementary Materials.

## Supplementary Materials

The following supporting information can be downloaded at https://www.mdpi.com/article/10.3390/md22040174/s1, Figure S1. CT induced the cell death of different cell lines. Figure S2. CT induced the cell death of A549 cells in a concentration- and time-dependent manner. Figure S3. STS induced the cell death of A549 cells. Figure S4. Western blot of cleaved-PARP and cleaved-caspase-3 in A549 cells. Figure S5. RNA-seq analysis of 10 µM CT-treated A549 cells. Figure S6. Western blot of p-IRE1α and LC3 II levels in A549 cell lines treated with CT.

## References

[1] Savić M.P., Sakač M.N., Kuzminac I.Z., Ajduković J.J. (2022). Structural diversity of bioactive steroid compounds isolated from soft corals in the period 2015–2020. J. Steroid Biochem. Mol. Biol..

[2] Carroll A.R., Copp B.R., Davis R.A., Keyzers R.A., Prinsep M.R. (2023). Marine natural products. Nat. Prod. Rep..

[3] Wang Z., Tang H., Wang P., Gong W., Xue M., Zhang H., Liu T., Liu B., Yi Y., Zhang W. (2013). Bioactive polyoxygenated steroids from the South China sea soft coral, *Sarcophyton* sp. Mar. Drugs.

[4] Liu T.-F., Lu X., Tang H., Zhang M.-M., Wang P., Sun P., Liu Z.-Y., Wang Z.-L., Li L., Rui Y.-C. (2013). 3β,5α,6β-Oxygenated sterols from the South China Sea gorgonian *Muriceopsis flavida* and their tumor cell growth inhibitory activity and apoptosis-inducing function. Steroids.

[5] Wang P., Tang H., Liu B.S., Li T.J., Sun P., Zhu W., Luo Y.P., Zhang W. (2013). Tumor cell growth inhibitory activity and structure-activity relationship of polyoxygenated steroids from the gorgonian *Menella kanisa*. Steroids.

[6] Xu C., Li J., Su L., Tang H., Zhang W. (2020). Osteoclastogenesis modulatory steroids from the South China Sea gorgonian coral *Iciligorgia* sp. Chem. Biodivers..

[7] Luoma P.V. (2007). Cytochrome P450—physiological key factor against cholesterol accumulation and the atherosclerotic vascular process. Ann. Med..

[8] Iuliano L. (2011). Pathways of cholesterol oxidation via non-enzymatic mechanisms. Chem. Phys. Lipids.

[9] de Medina P., Paillasse M.R., Segala G., Poirot M., Silvente-Poirot S. (2010). Identification and pharmacological characterization of cholesterol-5,6-epoxide hydrolase as a target for tamoxifen and AEBS ligands. Proc. Natl. Acad. Sci. USA.

[10] Zmysłowski A., Szterk A. (2019). Oxysterols as a biomarker in diseases. Clin. Chim. Acta.

[11] Samadi A., Sabuncuoglu S., Samadi M., Isikhan S.Y., Chirumbolo S., Peana M., Lay I., Yalcinkaya A., Bjørklund G. (2021). A Comprehensive review on oxysterols and related diseases. Curr. Med. Chem..

[12] de Freitas F.A., Levy D., Zarrouk A., Lizard G., Bydlowski S.P. (2021). Impact of oxysterols on cell death, proliferation, and differentiation induction: Current status. Cells.

[13] Nury T., Yammine A., Ghzaiel I., Sassi K., Zarrouk A., Brahmi F., Samadi M., Rup-Jacques S., Vervandier-Fasseur D., Pais de Barros J.P. (2021). Attenuation of 7-ketocholesterol- and 7β-hydroxycholesterol-induced oxiapoptophagy by nutrients, synthetic molecules and oils: Potential for the prevention of age-related diseases. Ageing Res. Rev..

[14] Pedruzzi E., Guichard C., Ollivier V., Driss F., Fay M., Prunet C., Marie J.C., Pouzet C., Samadi M., Elbim C. (2004). NAD(P)H oxidase Nox-4 mediates 7-ketocholesterol-induced endoplasmic reticulum stress and apoptosis in human aortic smooth muscle cells. Mol. Cell Biol..

[15] Prunet C., Lemaire-Ewing S., Ménétrier F., Néel D., Lizard G. (2005). Activation of caspase-3-dependent and -independent pathways during 7-ketocholesterol- and 7β-hydroxycholesterol-induced cell death: A morphological and biochemical study. J. Biochem. Mol. Toxicol..

[16] Monier S., Samadi M., Prunet C., Denance M., Laubriet A., Athias A., Berthier A., Steinmetz E., Jürgens G., Nègre-Salvayre A. (2003). Impairment of the cytotoxic and oxidative activities of 7β-hydroxycholesterol and 7-ketocholesterol by esterification with oleate. Biochem. Biophys. Res. Commun..

[17] Seye C.I., Knaapen M.W.M., Daret D., Desgranges C., Herman A.G., Kockx M.A., Bult H. (2004). 7-ketocholesterol induces reversible cytochrome c release in smooth muscle cells in absence of mitochondrial swelling. Cardiovasc. Res..

[18] Helmschrodt C., Becker S., Schröter J., Hecht M., Aust G., Thiery J., Ceglarek U. (2013). Fast LC–MS/MS analysis of free oxysterols derived from reactive oxygen species in human plasma and carotid plaque. Clin. Chim. Acta.

[19] Cheng Y.W., Kang J.J., Shih Y.L., Lo Y.L., Wang C.F. (2005). Cholesterol-3β,5α,6β-triol induced genotoxicity through reactive oxygen species formation. Food Chem. Toxicol..

[20] Liao P.L., Cheng Y.W., Li C.H., Lo Y.L., Kang J.J. (2009). Cholesterol-3β,5α,6β-triol induced PI(3)K-Akt-eNOS-dependent cyclooxygenase-2 expression in endothelial cells. Toxicol. Lett..

[21] Attanzio A., Frazzitta A., Cilla A., Livrea M.A., Tesoriere L., Allegra M. (2019). 7-Ketocholesterol and cholestane-3β,5α,6β-Triol induce eryptosis through distinct pathways leading to NADPH oxidase and nitric oxide synthase activation. Cell Physiol. Biochem..

[22] Liu H., Wang T., Huang K. (2009). Cholestane-3β,5α,6β-triol-induced reactive oxygen species production promotes mitochondrial dysfunction in isolated mice liver mitochondria. Chem. Biol. Interact..

[23] Liu H., Yuan L., Xu S., Wang K., Zhang T. (2005). Cholestane-3β,5α,6β-triol inhibits osteoblastic differentiation and promotes apoptosis of rat bone marrow stromal cells. J. Cell Biochem..

[24] Liu H.M., Yuan L., Xu S.J., Zhang T.L., Wang K. (2004). Cholestane-3β,5α,6β-triol promotes vascular smooth muscle cells calcification. Life Sci..

[25] Carvalho J.F.S.M.M., Moreira J.N., Joao N., Simoes S., Luisa Sa e Melo M. (2010). Sterols as anticancer agents: Synthesis of ring-B oxygenated steroids, cytotoxic profile, and comprehensive SAR analysis. J. Med. Chem..

[26] Liu H., Zhang C., Huang K. (2011). Lanthanum chloride suppresses oxysterol-induced ECV-304 cell apoptosis via inhibition of intracellular Ca^2+^ concentration elevation, oxidative stress, and activation of ERK and NF-κB signaling pathways. J. Biol. Inorg. Chem..

[27] Levy D., Correa de Melo T., Ohira B.Y., Fidelis M.L., Ruiz J.L.M., Rodrigues A., Bydlowski S.P. (2018). Oxysterols selectively promote short-term apoptosis in tumor cell lines. Biochem. Biophys. Res. Commun..

[28] Hu H., Tian M., Ding C., Yu S. (2018). The C/EBP homologous protein (CHOP) transcription factor functions in endoplasmic reticulum stress-induced apoptosis and microbial infection. Front. Immunol..

[29] Oyadomari S., Mori M. (2004). Roles of CHOP/GADD153 in endoplasmic reticulum stress. Cell Death Differ..

[30] Stein D., Slobodnik Z., Tam B., Einav M., Akabayov B., Berstein S., Toiber D. (2022). 4-Phenylbutyric acid—Identity crisis; can it act as a translation inhibitor?. Aging Cell.

[31] Urano F., Wang X., Bertolotti A., Zhang Y., Chung P., Harding H.P., Ron D. (2000). Coupling of stress in the ER to activation of JNK protein kinases by transmembrane protein kinase IRE1. Science.

[32] Kang R., Zeh H.J., Lotze M.T., Tang D. (2011). The Beclin 1 network regulates autophagy and apoptosis. Cell Death Differ..

[33] Tanida I., Ueno T., Kominami E. (2008). LC3 and autophagy. Methods Mol. Biol..

[34] Liu L., Han C., Yu H., Zhu W., Cui H., Zheng L., Zhang C., Yue L. (2018). Chloroquine inhibits cell growth in human A549 lung cancer cells by blocking autophagy and inducing mitochondrial-mediated apoptosis. Oncol. Rep..

[35] Moscat J., Diaz-Meco M.T. (2009). p62 at the Crossroads of autophagy, apoptosis, and cancer. Cell.

[36] Ye X., Zhou X.J., Zhang H. (2018). Exploring the role of autophagy-related gene 5 (ATG5) yields important insights into autophagy in autoimmune/autoinflammatory diseases. Front. Immunol..

[37] Testa G., Staurenghi E., Zerbinati C., Gargiulo S., Iuliano L., Giaccone G., Fanto F., Poli G., Leonarduzzi G., Gamba P. (2016). Changes in brain oxysterols at different stages of Alzheimer’s disease: Their involvement in neuroinflammation. Redox Biol..

[38] Kreilaus F., Spiro A.S., McLean C.A., Garner B., Jenner A.M. (2016). Evidence for altered cholesterol metabolism in Huntington’s disease post mortem brain tissue. Neuropathol. Appl. Neurobiol..

[39] Iuliano L., Micheletta F., Natoli S., Ginanni Corradini S., Iappelli M., Elisei W., Giovannelli L., Violi F., Diczfalusy U. (2003). Measurement of oxysterols and α-tocopherol in plasma and tissue samples as indices of oxidant stress status. Anal. Biochem..

[40] Raselli T., Hearn T., Wyss A., Atrott K., Peter A., Frey-Wagner I., Spalinger M.R., Maggio E.M., Sailer A.W., Schmitt J. (2019). Elevated oxysterol levels in human and mouse livers reflect nonalcoholic steatohepatitis. J. Lipid Res..

[41] Linseisen J., Wolfram G.N., Miller A.B. (2002). Plasma 7β-hydroxycholesterol as a possible predictor of lung cancer risk. Cancer Epidemiol. Biomark. Prev..

[42] Kloudova-Spalenkova A., Ueng Y.-F., Wei S., Kopeckova K., Guengerich F.P., Soucek P. (2020). Plasma oxysterol levels in luminal subtype breast cancer patients are associated with clinical data. J. Steroid Biochem. Mol. Biol..

[43] Reddy B.S., Martin C.W., Wynder E.L. (1977). Fecal bile acids and cholesterol metabolites of patients with ulcerative colitis, a high-risk group for development of colon cancer. Cancer Res..

[44] Lin C.Y., Huo C., Kuo L.K., Hiipakka R.A., Jones R.B., Lin H.P., Hung Y., Su L.C., Tseng J.C., Kuo Y.Y. (2013). Cholestane-3β,5α,6β-triol suppresses proliferation, migration, and invasion of human prostate cancer cells. PLoS ONE.

[45] Jusakul A., Loilome W., Namwat N., Haigh W.G., Kuver R., Dechakhamphu S., Sukontawarin P., Pinlaor S., Lee S.P., Yongvanit P. (2012). Liver fluke-induced hepatic oxysterols stimulate DNA damage and apoptosis in cultured human cholangiocytes. Mutat. Res..

[46] Wu Q.Z., Huang K.X. (2006). Protective effect of ebselen on cytotoxicity induced by cholestane-3β,5α,6β-triol in ECV-304 cells. Biochim. Biophys. Acta.

[47] Zhang J., Guo J., Yang N., Huang Y., Hu T., Rao C. (2022). Endoplasmic reticulum stress-mediated cell death in liver injury. Cell Death Dis..

[48] Han J., Cheng C., Zhang J., Fang J., Yao W., Zhu Y., Xiu Z., Jin N., Lu H., Li X. (2022). Myricetin activates the caspase-3/GSDME pathway via ER stress induction of pyroptosis in lung cancer cells. Front. Pharmacol..

[49] Kaspar S., Oertlin C., Szczepanowska K., Kukat A., Senft K., Lucas C., Brodesser S., Hatzoglou M., Larsson O., Topisirovic I. (2021). Adaptation to mitochondrial stress requires CHOP-directed tuning of ISR. Sci. Adv..

[50] Liu S., Yao S., Yang H., Liu S., Wang Y. (2023). Autophagy: Regulator of cell death. Cell Death Dis..

[51] Denton D., Kumar S. (2019). Autophagy-dependent cell death. Cell Death Differ..

[52] Vejux A., Abed-Vieillard D., Hajji K., Zarrouk A., Mackrill J.J., Ghosh S., Nury T., Yammine A., Zaibi M., Mihoubi W. (2020). 7-Ketocholesterol and 7β-hydroxycholesterol: In vitro and animal models used to characterize their activities and to identify molecules preventing their toxicity. Biochem. Pharmacol..

[53] Bellanti F., Villani R., Tamborra R., Blonda M., Iannelli G., di Bello G., Facciorusso A., Poli G., Iuliano L., Avolio C. (2018). Synergistic interaction of fatty acids and oxysterols impairs mitochondrial function and limits liver adaptation during nafld progression. Redox Biol..

[54] de Dios C., Abadin X., Roca-Agujetas V., Jimenez-Martinez M., Morales A., Trullas R., Mari M., Colell A. (2023). Inflammasome activation under high cholesterol load triggers a protective microglial phenotype while promoting neuronal pyroptosis. Transl. Neurodegener..

