# Supplementary material for: Cholestane-3β,5α,6β-triol Induces Multiple Cell Death in A549 Cells via ER Stress and Autophagy Activation
Source: Mar Drugs. 2024 Apr 13;22(4):174. doi: 10.3390/md22040174 (PMC11051220; doi:10.3390/md22040174)
Supplement: Supplementary file 1 [file marinedrugs-22-00174-s001.zip › marinedrugs-2917719 Supplementary Material.pdf]

Supplementary Material

Cholestane-3β,5a,6β-triol induces multiple cell death in A549 cells via ER stress and autophagy activation

Jiaxi Chen [1,†], Jieping Zhang [1,†], Lijuan Cai [1], Li Guo [1], Zhenyu Cai [1], Hua Han [1,*] and Wen Zhang [1,2,*]

1 School of Medicine, Tongji University, 1239 Si-Ping Road, Shanghai 200092, China

2 Ningbo Institute of Marine Medicine, Peking University, 56 Kang-Da Road, Ningbo 315832, China

* Correspondence: hanhua@tongji.edu.cn (H.H.); wenzhang1968@163.com (W.Z.)

† These authors contributed equally to this work.

Table of contents

Figure S1. CT induced the cell death of different cell lines. 2

Figure S2. CT induced the cell death of A549 cells at concentration- and time-dependent manner. 3

Figure S3. STS induced the cell death of A549 cells. 3

Figure S4. Western blot of cleaved-PARP and cleaved-caspase-3 in A549 cells.4

Figure S5. RNA-seq analysis of 10 μM CT-treated A549 cells. 4

Figure S6. Western blot of p-IRE1α and LC3 II levels in A549 cell lines treated with CT. 5

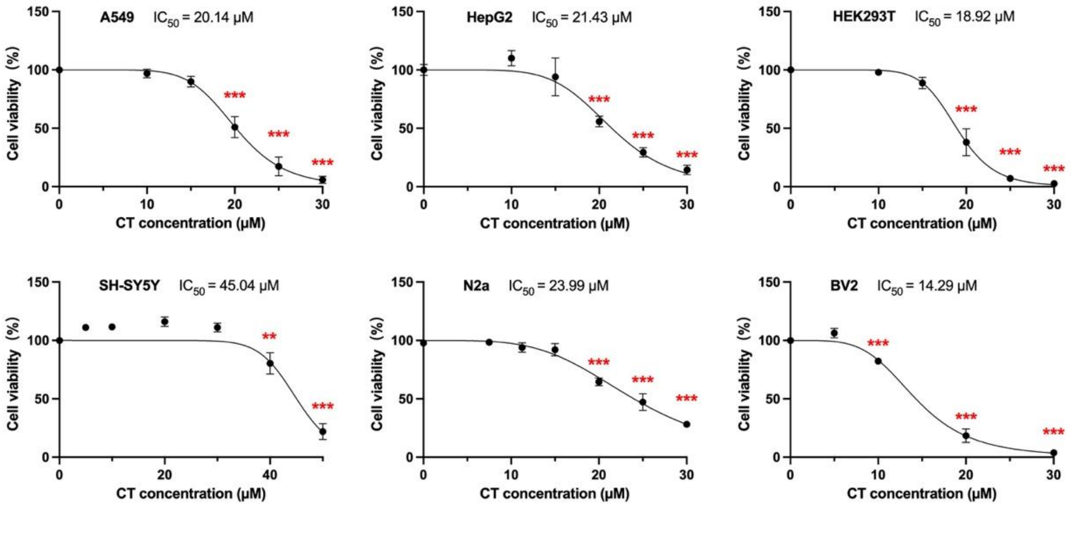

Figure S1. CT induced the cell death of different cell lines. CT inhibited the cell viability of A549, HepG2, HEK293T, SH-SY5Y, N2a, BV2 cells. Cell viability was assessed by CCK-8 kit after the treatment of CT at different concentrations for 24 h. Statistical significance was determined using one-way ANOVA with Dunnett's post-test, $^{**}p < 0.005$, $^{***}p < 0.001$ compared with the NC group.

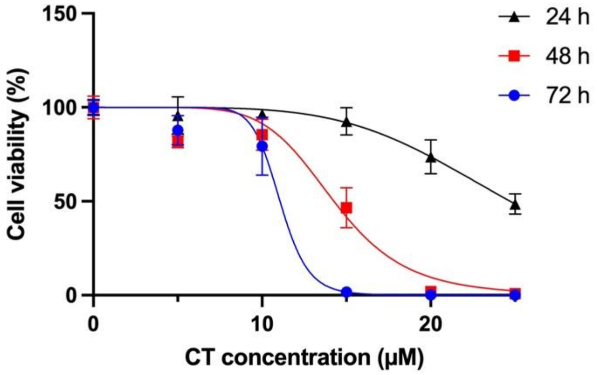

Figure S2. CT induced the cell death of A549 cells at concentration- and time-dependent manner. Cell viability was assessed by CCK-8 kit after the treatment of CT at different concentrations for 24, 48, 72 h.

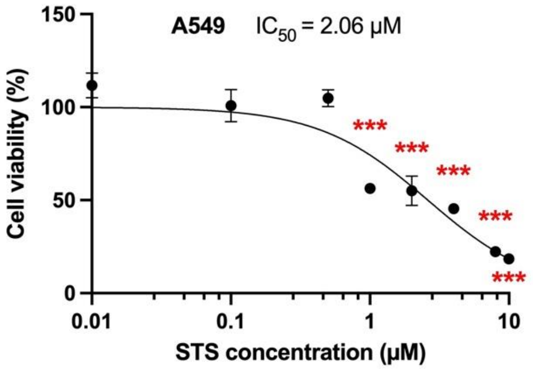

Figure S3. STS induced the cell death of A549 cells. Cell viability was assessed by CCK-8 kit after the treatment of STS at different concentrations for 24 h. Statistical significance was determined using one-way ANOVA with Dunnett's post-test, $n = 2$, ***$p < 0.001$ compared with the NC group.

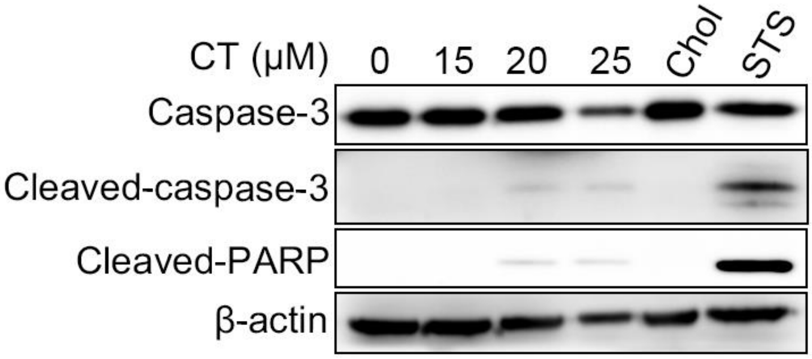

Figure S4. Western blot of cleaved-PARP and cleaved-caspase-3 in A549 cells. Cells were treated with series doses of CT for 24 h. Cells were treated with 1 μM STS for 24 h as the positive control, and 20 μM cholesterol (Chol) for 24 h as an isotype control.

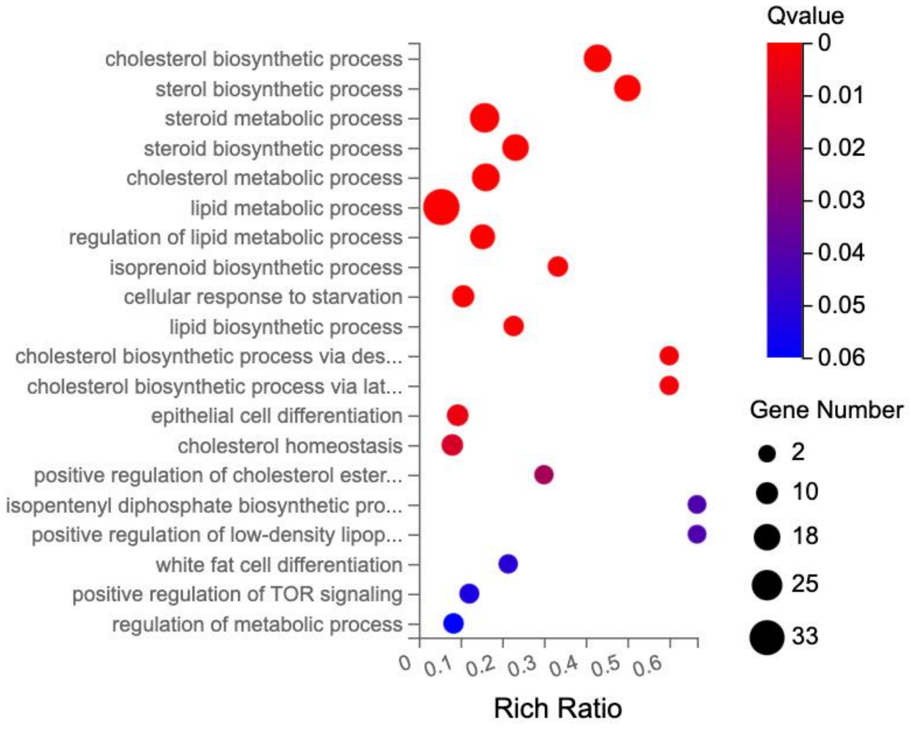

Figure S5. RNA-seq analysis of 10 μM CT-treated A549 cells. GO analysis of

the upregulated gene clusters in CT 10 μM treatment groups. The function of Q value ≤ 0.05 was considered as significant enrichment.

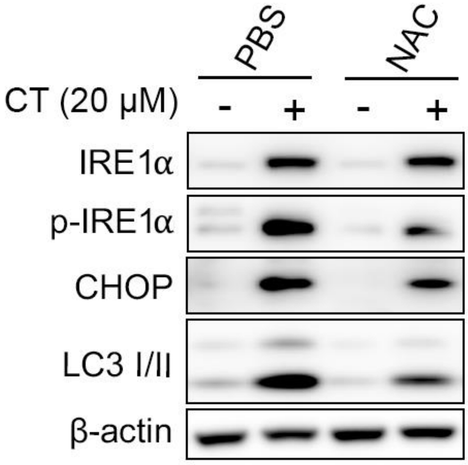

Figure S6. Western blot of p-IRE1α and LC3 II levels in A549 cell lines treated with CT. The cells were pre-treated with 1 mM 4-PBA or 3 mM NAC for 1 h, then incubated with CT at 20 μM for 24 h.
